# Author Correction: EXCRETE workflow enables deep proteomics of the microbial extracellular environment

**DOI:** 10.1038/s42003-026-10208-w

**Published:** 2026-05-15

**Authors:** David A. Russo, Denys Oliinyk, Georg Pohnert, Florian Meier, Julie A. Z. Zedler

**Affiliations:** 1 Bioorganic Analytics, Institute for Inorganic and Analytical Chemistry, Friedrich Schiller University Jena, Jena, Germany; 2 Functional Proteomics, Jena University Hospital, Jena, Germany; 3 Synthetic Biology of Photosynthetic Organisms, Matthias Schleiden Institute for Genetics, Bioinformatics and Molecular Botany, Friedrich Schiller University Jena, Jena, Germany

Correction to: *Communications Biology* 10.1038/s42003-024-06910-2, published online 25 September 2024

In the version of this article initially published, Fig. 3a–d mistakenly included a duplicated biological replicate. The figure has now been revised in the HTML and PDF versions of the article. For comparison, the original Fig. 3 is available as Supplementary Information accompanying this amendment.

## Supplementary information

Original Fig. 3

