# Supplementary material for: EXCRETE workflow enables deep proteomics of the microbial extracellular environment
Source: Commun Biol. 2024 Sep 25;7:1189. doi: 10.1038/s42003-024-06910-2 (PMC11424642; doi:10.1038/s42003-024-06910-2)
Supplement: Supplementary file 2 — Supplementary Information [file 42003_2024_6910_MOESM2_ESM.pdf]

Supplementary Information

EXCRETE workflow enables deep proteomics of the microbial extracellular environment

David A. Russo[1*†], Denys Oliinyk[2†], Georg Pohnert[1], Florian Meier[2], Julie A. Z. Zedler[3]

[1] Bioorganic Analytics, Institute for Inorganic and Analytical Chemistry, Friedrich Schiller University Jena, Jena, Germany
[2] Functional Proteomics, Jena University Hospital, Jena, Germany
[3] Synthetic Biology of Photosynthetic Organisms, Matthias Schleiden Institute for Genetics, Bioinformatics and Molecular Botany, Friedrich Schiller University Jena, Jena, Germany

[*] corresponding author, email: david.russo@uni-jena.de
[†] these authors contributed equally

Files included in this document:
Supplementary Figures 1-7
Supplementary Tables 1-5
Separate SI excel sheets given for Supplementary Data 1-15

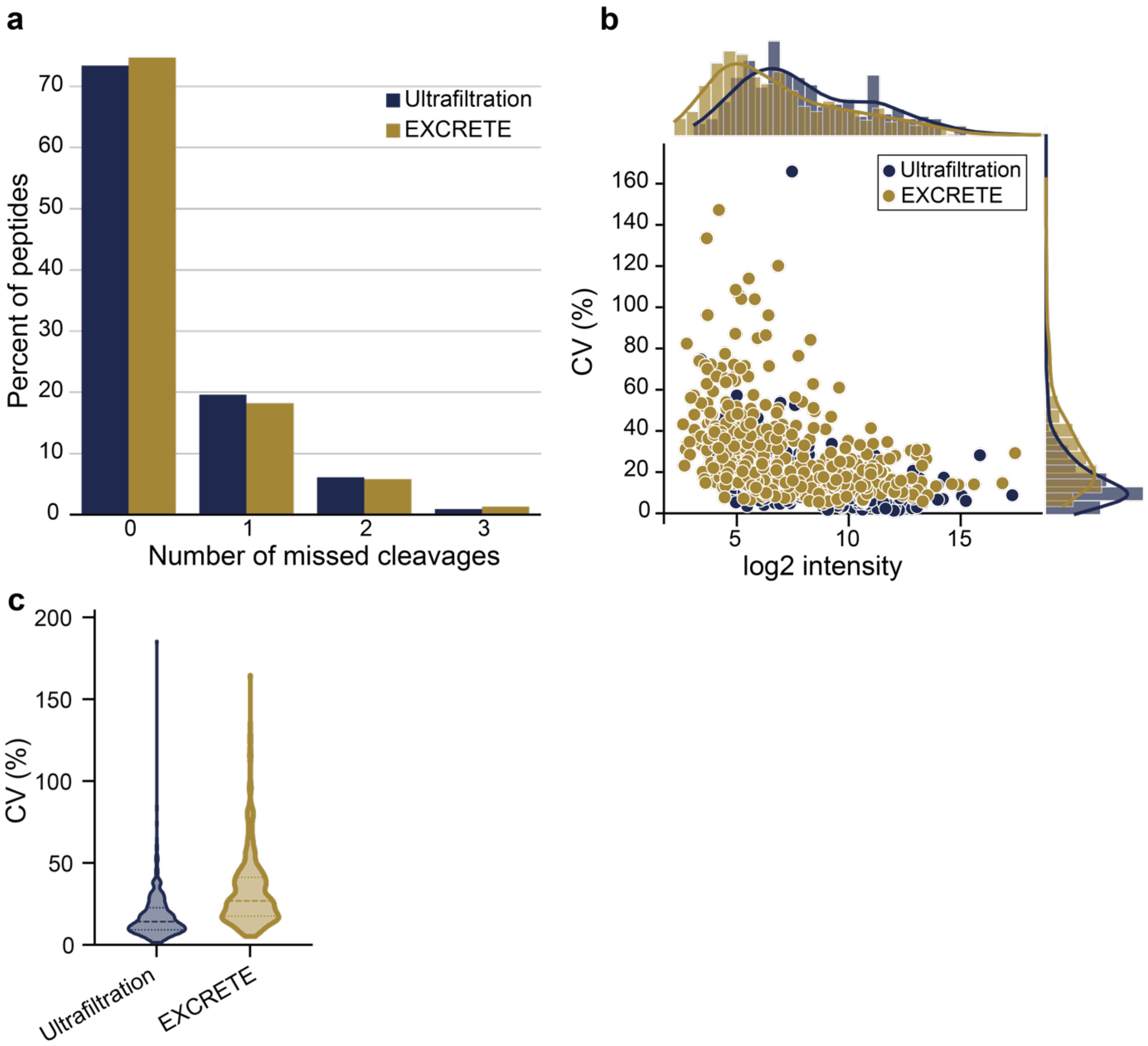

Supplementary Figure 1 Benchmarking of EXCRETE against ultrafiltration-based exoproteomic sample preparation. **a** Percentage of peptides containing missed cleavages after digestion with Trypsin and Lys-c **b** Coefficients of variation of the raw intensities of proteins (CVs) ordered by log2 protein intensity. On the secondary x-axis histogram and density plots representing the frequency distribution of protein intensities are shown. On the secondary y-axis histogram and density plots representing the frequency distribution of CVs are shown. Dots represent means of biological replicates. **c** CV of the raw intensities of proteins identified with ultrafiltration and EXCRETE. Dashed line represents the median. Dotted lines represent the top and bottom quartiles.

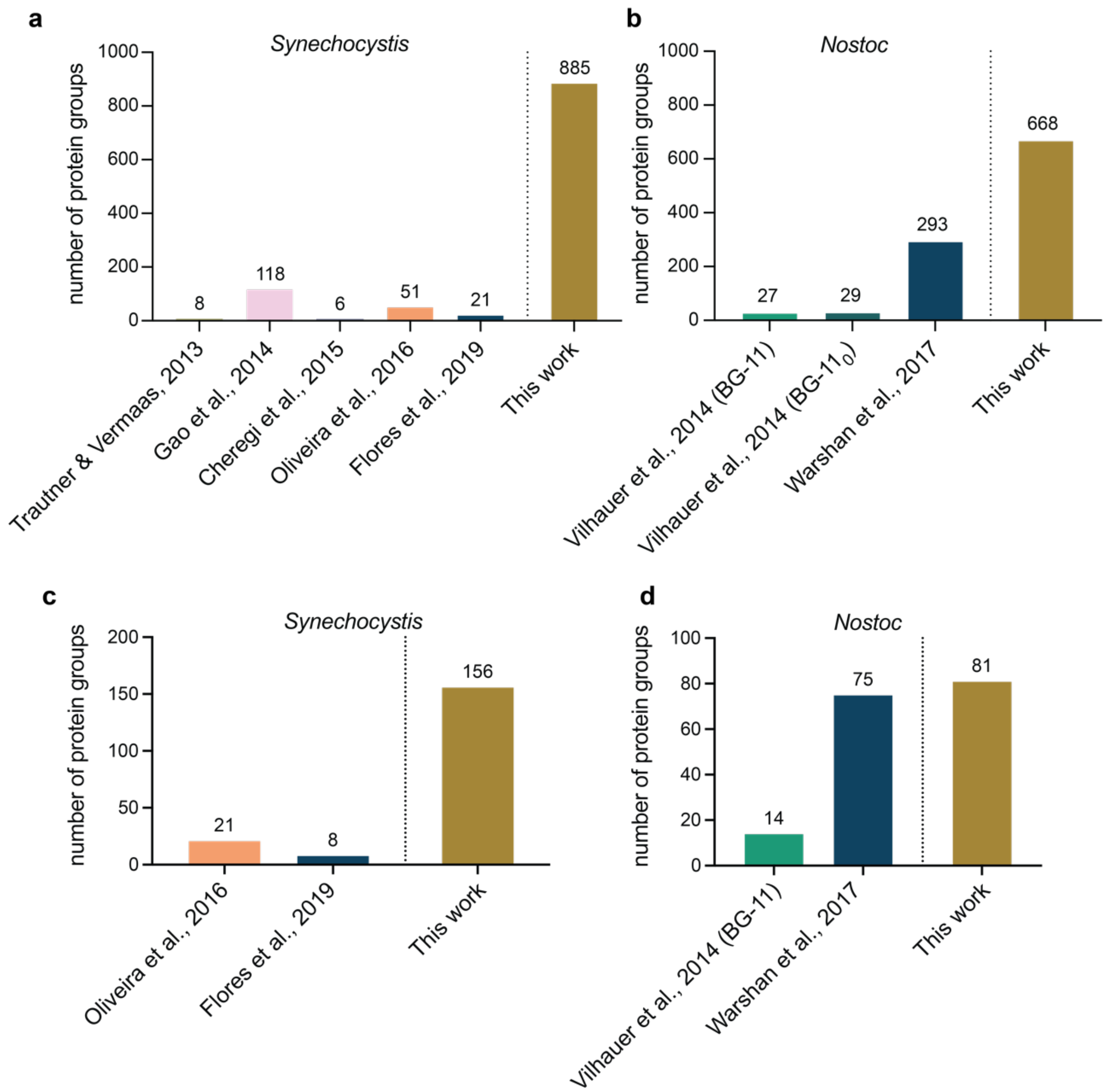

Supplementary Figure 2 Comparison between the number of proteins identified in existing studies with the number identified in this study with EXCRETE. **a**, **b** Number of proteins identified in the exoproteome of *Synechocystis* (**a**) and *Nostoc* (**b**). **c**, **d** Subset of proteins from the exoproteome *Synechocystis* (**c**) and *Nostoc* (**d**) predicted as secreted.

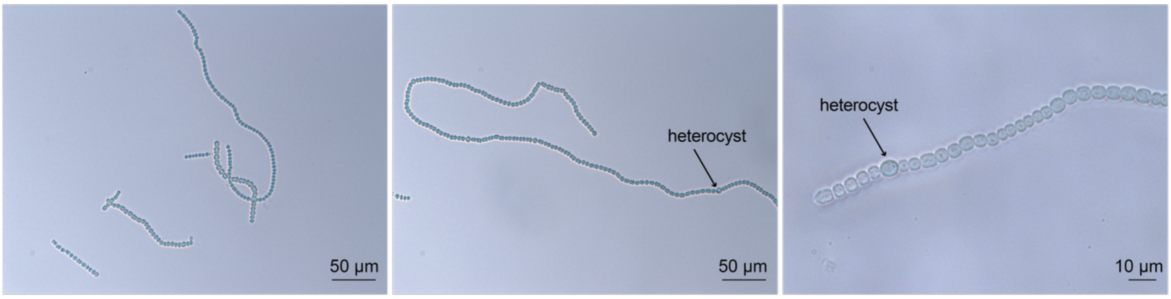

Supplementary Figure 3 Microscopic images of *Nostoc* at the point of harvesting for exoproteome analysis. Black arrows point at heterocysts. Left and center panels, scale bars 50 µm. Right panel, scale bar 10 µm.

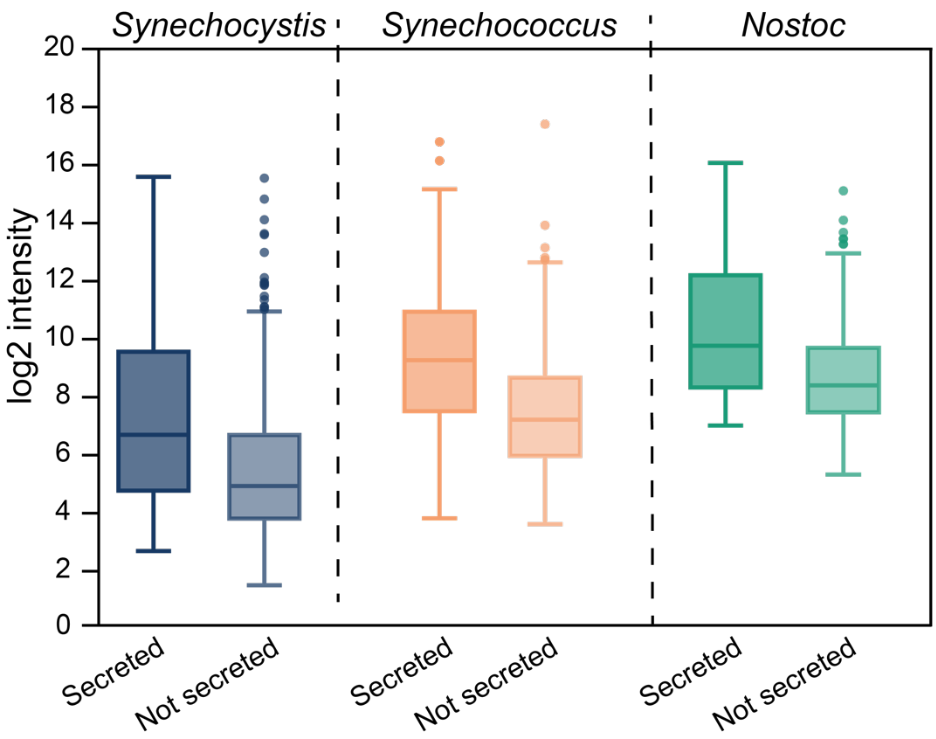

Supplementary Figure 4 Total intensity of secreted and non-secreted proteins identified in *Synechocystis*, *Synechococcus* and *Nostoc*. Centre line of boxplots, median; box limits, upper and lower quartiles; whiskers plotted according to Tukey method; dots, outliers.

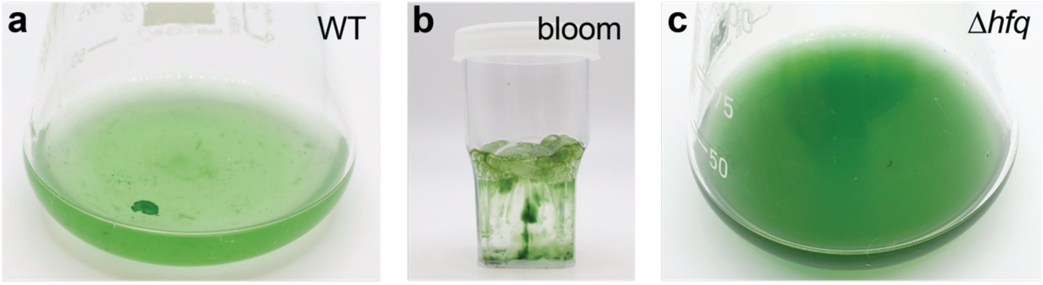

Supplementary Figure 5 Representative pictures of *Synechocystis* cultures. **a** WT in standard conditions. **b** Bloom-like culture cultivated in elevated CO_2. **c** Δ*hfq* mutant.

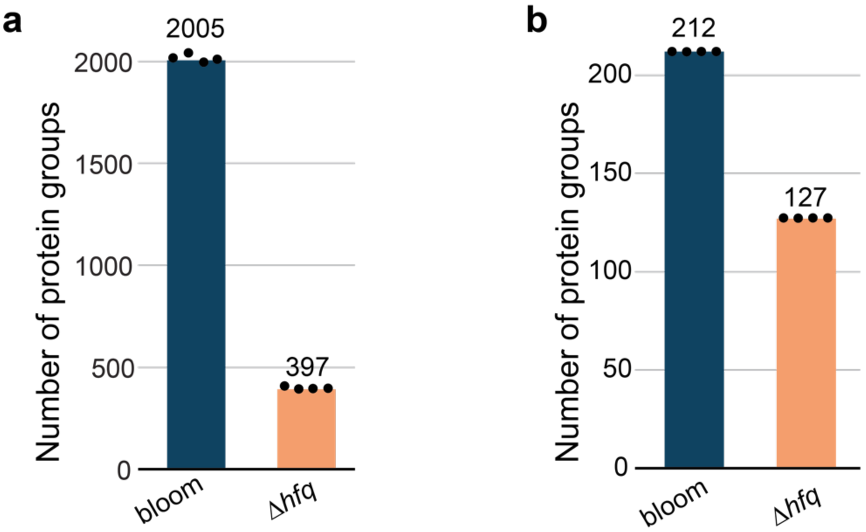

Supplementary Figure 6 Number of total and secreted protein groups, after filtering and imputation, in the *Synechocystis* exoproteome in different conditions. **a, b** Number of total (**a**) and secreted (**b**) protein groups identified in a bloom-like culture and a Δ*hfq* mutant. Means are shown above the bars. Black dots represent biological replicates (n = 4).

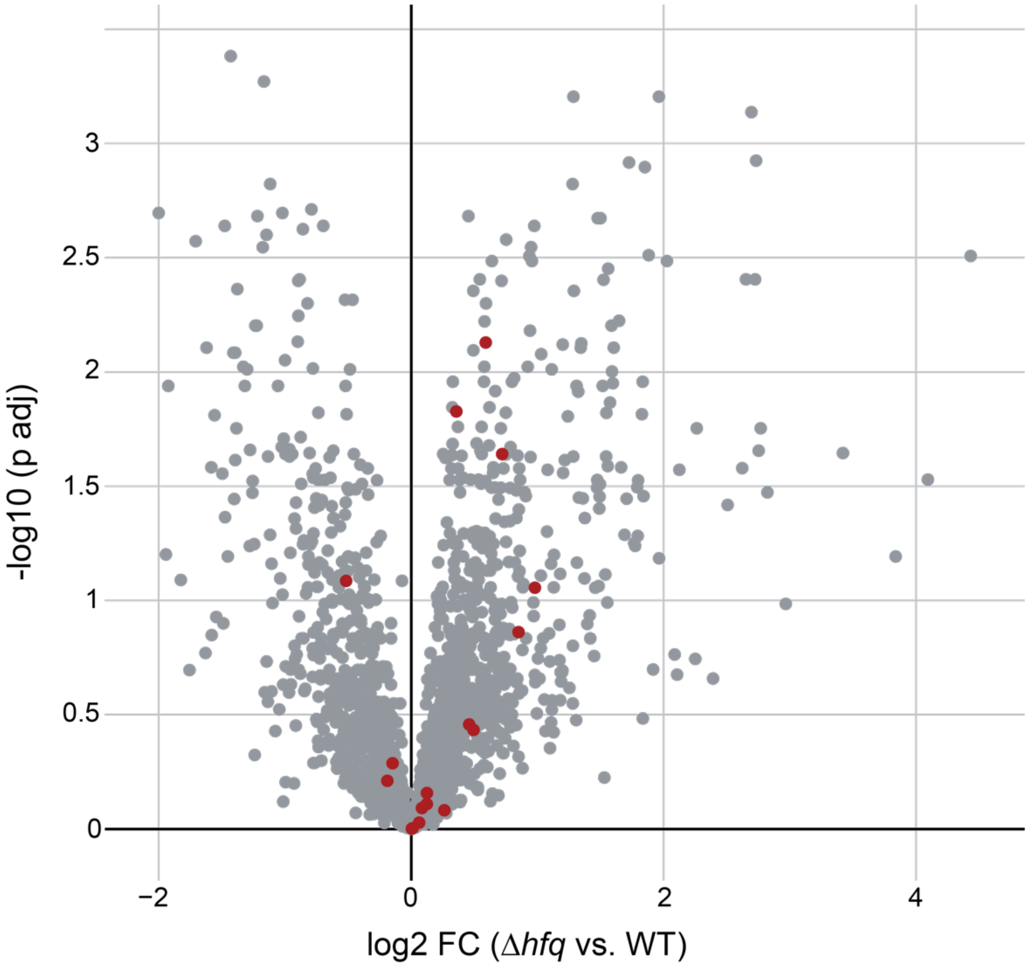

Supplementary Figure 7 Volcano plot illustrating differential protein expression between proteins identified in the endoproteomes of WT *Synechocystis* and the Δ*hfq* mutant. Each dot on the plot represents an individual protein. Red dots indicate proteins that are absent from the *Synechocystis* secretome in the Δ*hfq* condition when compared to the WT conditions.

Supplementary Table 1. Analysis of the percentage of intracellular marker proteins in the endoproteome of *Synechocystis* and *Nostoc* in comparison with the exoproteome of multiple cyanobacteria.

Species	Photosystem subunits	Phycobilins	Ribosomes	RuBisCO	Reference
Endoproteome					
Synechocystis sp. PCC 6803	12.1%	17.2%	4.7%	3.5%	This work
Nostoc punctiforme PCC 73102	6.2%	6.4%	15.9%	3.1%	This work
Exoproteome					
Synechocystis sp. PCC 6803	1.5%	5.9%	1.1%	0.1%	This work
Synechococcus sp. PCC 11901	0.3%	1.1%	0.0%	0.0%	This work
Nostoc punctiforme PCC 73102	0.6%	2.0%	6.0%	1.2%	This work
Synechococcus sp. WH 5701	3.0%	9.3%	1.4%	0.1%	Christie-Oleza et al., 2015
Synechococcus sp. WH 7803	2.3%	26.1%	0.6%	0.1%	Christie-Oleza et al., 2015
Synechococcus sp. WH 7805	0.0%	12.9%	0.0%	0.0%	Christie-Oleza et al., 2015
Synechococcus sp. WH 8102	2.8%	37.3%	1.1%	0.0%	Christie-Oleza et al., 2015

Supplementary Table 2. Putative T1SS substrates identified in the *Synechocystis* secretome.

Protein ID (NCBI)	Protein ID (UniprotKB)	Protein name	Length (AA)	Locus tag	PosrtB localization	Signal peptide	PI	Gly (%)	Cys (%)
AGF52383.1	NA	hypothetical protein	4787	NA	Extracellular	NA	3.43	9.4	0.1
AGF53432.1	Q6ZEX5	hypothetical protein	3797	slr5005	Extracellular	NA	4.41	11.7	0
AGF52230.1	P74440	integrin alpha subunit domain-like protein	4199	slr0408	Extracellular	NA	3.99	10.9	0
AGF52739.1	Q55365	endo-1,4-beta-glucanase	1070	slr0897	Extracellular	NA	4.6	13.7	0
AGF53312.1	P74647	hypothetical protein	1771	sll0723	Extracellular	NA	4.37	10.9	0
AGF53314.1	P74649	leukotoxin LtA	1290	sll0721	Extracellular	NA	4.03	12.8	0
AGF50742.1	P73032	hypothetical protein	1749	slr1753	Extracellular	Sec lipo	3.81	10.2	0.2
AGF50645.1	P72939	alkaline phosphatase	1409	sll0654	Periplasm	NA	3.93	9.9	0
AGF51323.1	P73590	integrin alpha- and beta4-subunit domain-like protein	3016	slr1403	Extracellular	NA	3.98	14.7	0
AGF53129.1	Q55489	hypothetical protein	948	sll0499	Outer membrane	NA	4.6	6.9	0.1
AGF50804.1	P73089	fat protein	1965	slr2046	Extracellular	NA	3.61	7.8	0

Supplementary Table 3. Reciprocal best hits BLAST analysis of the proteins identified in the top COG categories in *Synechocystis*, *Synechococcus* and *Nostoc*. Percentages represent the number of proteins in each category of each species that have an ortholog in the same category of the second species.

**% orthologs**	**M**	**P**	**O**	**S**
Synechocystis:Synechococcus	53%	17%	30%	24%
Synechocystis:Nostoc	35%	50%	50%	22%
Synechococcus:Synechocystis	47%	8%	60%	20%
Synechococcus:Nostoc	47%	8%	80%	16%
Nostoc:Synechocystis	35%	43%	63%	45%
Nostoc:Synechococcus	53%	14%	50%	40%
average	45%	23%	55%	28%

Supplementary Table 4. COG classification of the 54 proteins upregulated in the *Synechocystis* secretome in the Δ*hfq* condition in comparison to the WT condition.

COG category	%
Not attributed	30
Cell envelope biogenesis	19
Function unknown	13
Carbohydrate transport and metabolism	9
Secondary metabolism	9
Signal transduction mechanism	9
Trafficking, secretion, and vesicular transport	6
Amino acid transport and metabolism	4
Cell cycle control and cell division	4
Transcription	2
Replication, recombination and repair	2
Energy production and conversion	2
Defense mechanisms	2
Inorganic ion transport and metabolism	2

Supplementary Table 5. Proteins identified in the reciprocal best hits BLAST analysis with orthologs in all three species.

Synechococcus		***Synechocystis***		***Nostoc***	
M					
A0A4P8WYT4	N-acetylmuramoyl-L-alanine amidase	P73736	N-acetylmuramoyl-L-alanine amidase	B2J2S4	Cell wall hydrolase/autolysin (EC 3.5.1.28)
A0A4P8WXS8	S-layer protein	P73409	hypothetical protein	B2IVV3	Carbohydrate-selective porin OprB
A0A4P8WY43	POTRA domain-containing protein	P73472	IaP75	B2IWK5	Surface antigen (D15)
A0A4P8X5N6	DUF3769 domain-containing protein	Q55580	hypothetical protein	B2J265	OstA family protein
O					
A0A4P8WZY2	Peptidase S8 and S53 subtilisin kexin sedolisin	P74698	hypothetical protein	B2J0L2	Peptidase S8 and S53, subtilisin, kexin, sedolisin
A0A4P8X170	Trypsin-like serine protease	P72780	protease HhoA	B2J9T3	Peptidase S1 and S6, chymotrypsin/Hap
A0A4V1G215	Trypsin-like serine protease	P73940	protease HhoB	B2J1D5	Peptidase S1 and S6, chymotrypsin/Hap
S					
A0A4P8X1D6	MBL fold metallo-hydrolase	P73222	hypothetical protein	B2IYH2	Zn-dependent hydrolase of the beta-lactamase fold-like protein
A0A4P8X2T3	Metallophosphoesterase	P72715	alkaline phosphatase	B2IV58	Metallophosphoesterase (EC 3.1.3.2)
A0A4P8X533	Tetratricopeptide repeat protein	P72802	outer membrane 72K protein	B2J928	TPR repeat-containing protein
A0A4P8X575	Pentapeptide repeat-containing protein	P74725	hypothetical protein	B2J260	Pentapeptide repeat protein

References

1. Cheregi, O., Miranda, H., Gröbner, G. & Funk, C. Inactivation of the Deg protease family in the cyanobacterium *Synechocystis* sp. PCC 6803 has impact on the outer cell layers. *Journal of Photochemistry and Photobiology B: Biology* **152**, 383–394 (2015).

2. Christie-Oleza, J. A., Armengaud, J., Guerin, P. & Scanlan, D. J. Functional distinctness in the exoproteomes of marine *Synechococcus*. *Environmental Microbiology* **17**, 3781–3794 (2015).

3. Flores, C. *et al.* The alternative sigma factor SigF is a key player in the control of secretion mechanisms in *Synechocystis* sp. PCC 6803. *Environ Microbiol* **21**, 343–359 (2019).

4. Gao, L. *et al.* Profiling and compositional analysis of the exoproteome of *Synechocystis* sp. PCC 6803. *J Metabolomics Syst Biol* **1**, 8 (2014).

5. Oliveira, P. *et al.* The versatile TolC-like Slr1270 in the cyanobacterium *Synechocystis* sp. PCC 6803. *Environ Microbiol* **18**, 486–502 (2016).

6. Trautner, C. & Vermaas, W. F. J. The sll1951 gene encodes the surface layer protein of *Synechocystis* sp. strain PCC 6803. J Bacteriol 195, 5370–5380 (2013).

7. Vilhauer, L., Jervis, J., Ray, W. K. & Helm, R. F. The exo-proteome and exo-metabolome of *Nostoc punctiforme* (Cyanobacteria) in the presence and absence of nitrate. Arch Microbiol 196, 357–367 (2014).

8. Warshan, D. *et al.* Feathermoss and epiphytic *Nostoc* cooperate differently: Expanding the spectrum of plant–cyanobacteria symbiosis. *ISME J* **11**, 2821–2833 (2017).
