# Supplementary material for: EXCRETE workflow enables deep proteomics of the microbial extracellular environment
Source: Commun Biol. 2024 Sep 25;7:1189. doi: 10.1038/s42003-024-06910-2 (PMC11424642; doi:10.1038/s42003-024-06910-2)
Supplement: Supplementary file 4 — Description of Additional Supplementary Files [file 42003_2024_6910_MOESM4_ESM.pdf]

Description of Additional Supplementary Files

File name: Supplementary Data 1

Description: List of proteins identified in the exoproteome of Synechococcus sp. PCC 11901 with the EXCRETE workflow

File name: Supplementary Data 2

Description: List of proteins identified in the exoproteome of Synechococcus sp. PCC 11901 with the ultrafiltration workflow

File name: Supplementary Data 3

Description: List of proteins unique to the EXCRETE workflow

File name: Supplementary Data 4

Description: List of proteins identified in the exoproteome of Synechococcus sp. PCC 11901 processed in microtubes following filtration and imputation

File name: Supplementary Data 5

Description: List of proteins identified in the exoproteome of Synechococcus sp. PCC 11901 processed in microplates following filtration and imputation

File name: Supplementary Data 6

Description: List of proteins unique to the EXCRETE microtube workflow

File name: Supplementary Data 7

Description: List of proteins unique to the EXCRETE microplate workflow

File name: Supplementary Data 8

Description: List of proteins identified in the exoproteome of Synechocystis sp. PCC 6803 with the EXCRETE workflow

File name: Supplementary Data 9

Description: List of proteins identified in the exoproteome of Nostoc punctiforme PCC 73102 with the EXCRETE workflow

File name: Supplementary Data 10

Description: List of proteins identified in the endoproteome of Synechocystis sp. PCC 6803 with the EXCRETE workflow

File name: Supplementary Data 11

Description: List of proteins identified in the endoproteome of Nostoc punctiforme PCC 73102 with the EXCRETE workflow

File name: Supplementary Data 12

Description: List of proteins identified in the exoproteome of Synechocystis sp. PCC 6803 in the bloom condition with the EXCRETE workflow

File name: Supplementary Data 13

Description: List of proteins identified in the exoproteome of Synechocystis sp. PCC 6803 in the Δhfq condition with the EXCRETE workflow

File name: Supplementary Data 14

Description: List of proteins unique to the Synechocystis sp. PCC 6803 WT condition in comparison to the Δhfq condition

File name: Supplementary Data 15

Description: List of proteins identified in the endoproteome of Synechocystis sp. PCC 6803 Δhfq with the EXCRETE workflow
