# Supplementary figures and images for: Author Correction: EXCRETE workflow enables deep proteomics of the microbial extracellular environment
Source: Commun Biol. 2026 May 15;9:662. doi: 10.1038/s42003-026-10208-w (PMC13179312; doi:10.1038/s42003-026-10208-w)

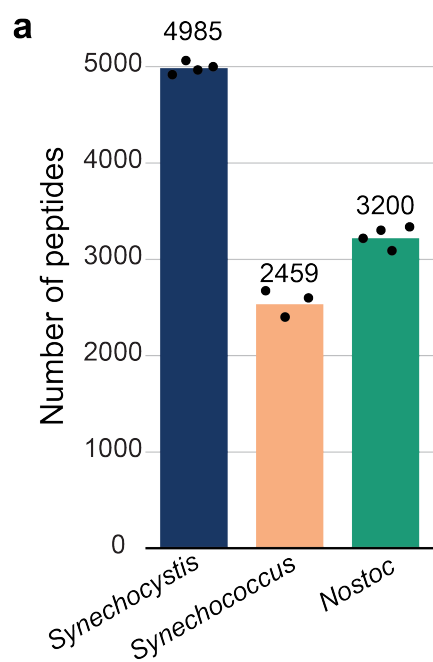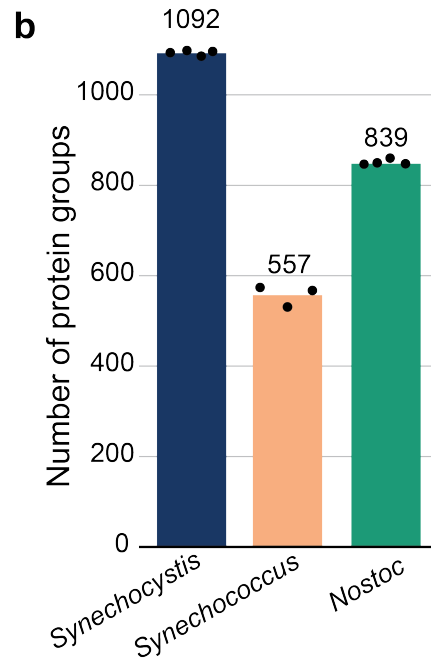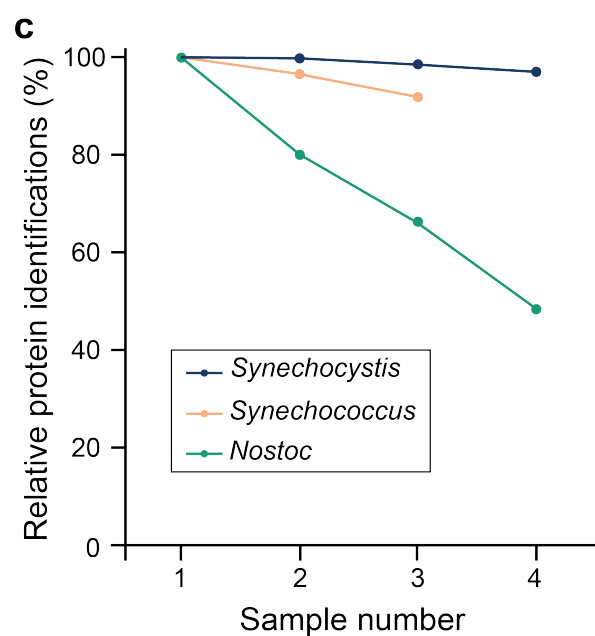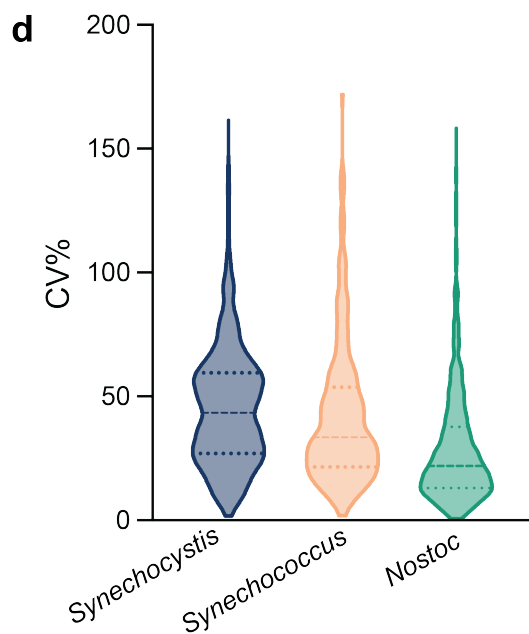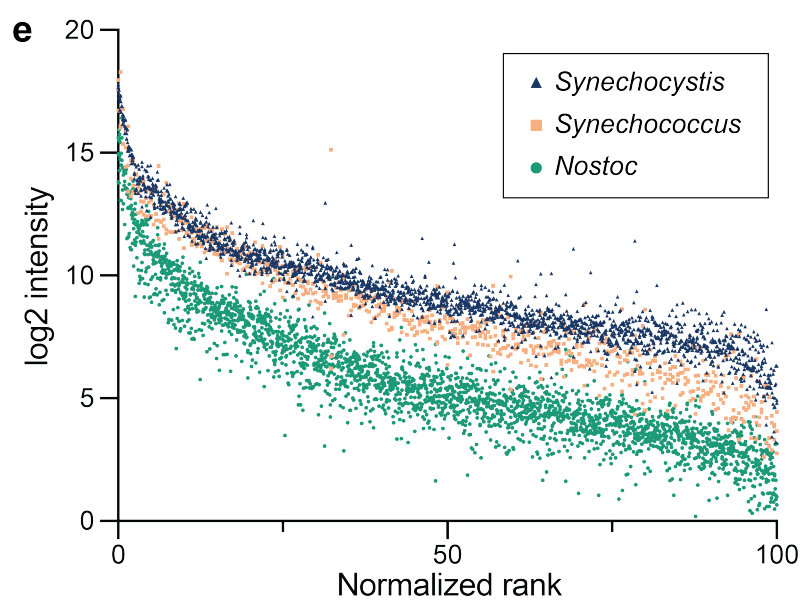

Supplement: Supplementary file 1 — Original Fig. 3 [file 42003_2026_10208_MOESM1_ESM.pdf]
